# Colonic Ischemia in a Patient With Pemphigus Vulgaris: Coincidence or Causation

**DOI:** 10.7759/cureus.110731

**Published:** 2026-06-12

**Authors:** Raveena Joshi, Deki Tsering, Emily Krueger, Natalie Wall, Munazza Anis, Thomas A Miller, Stephen Cohen

**Affiliations:** 1 Orthopaedics, Virginia Commonwealth University School of Medicine, Richmond, USA; 2 Surgery, Virginia Commonwealth University, Richmond, USA; 3 Radiology, Central Virginia VA Health Care System, Richmond, USA; 4 Surgery, Central Virginia VA Health Care System, Richmond, USA; 5 General and Colorectal Surgery, Central Virginia VA Health Care System, Richmond, USA

**Keywords:** bowel obstuction, colonic ischemia, corticosteroids, intravenous immunoglobulin, pemphigus vulgaris

## Abstract

Pemphigus vulgaris (PV) is a rare autoimmune blistering disorder requiring immunosuppression therapy for optimal treatment. We report the case of a 68-year-old man with newly diagnosed PV who developed colonic ischemia while undergoing treatment with corticosteroids and intravenous immunoglobulin (IVIG). The “acute abdomen” that developed from this ischemia mandated abdominal exploration, resulting in ileocecectomy for effective management. To our knowledge, this is the first documented case of colonic ischemia temporally associated with IVIG therapy in a patient with pemphigus vulgaris. This case highlights the clinical relevance of recognizing the potential for gastrointestinal complications and emphasizing the importance of vigilance while treating PV patients with IVIG.

## Introduction

Pemphigus vulgaris (PV) is a rare autoimmune blistering disorder caused by autoantibodies directed against desmoglein 3, the "glue" holding cells together, resulting in acantholysis and blistering of mucosal and cutaneous surfaces [1]. Such lesions are classically flaccid, superficial blisters that rapidly rupture, resulting in erosions and crusting. Mucosal erosions, most commonly in the oral cavity, are present in about 90% of patients at some point during the disease course, with the primary symptom reported as pain. The pain can be so severe that it adversely affects chewing and swallowing, thereby affecting nutrition. Exclusive oral mucosal involvement occurs in approximately 50.8% of cases at initial presentation [2]. Pruritus is less common than pain but may occur, especially in cutaneous forms. The Nikolsky sign (epidermal detachment upon lateral pressure) is often positive in affected areas. The annual incidence is estimated at 0.1-2.7 cases per 100,000 persons [3]. Current management includes systemic corticosteroids and rituximab, a powerful B-cell-targeting biologic that can induce remission.

Against this background, we report a case of a 68-year-old man with newly diagnosed PV who developed acute colonic ischemia shortly after initiation of treatment with high-dose corticosteroids and intravenous immunoglobulin (IVIG). His clinical deterioration required urgent exploratory laparotomy and ileocecectomy. This case highlights a rare but potentially life-threatening gastrointestinal (GI) complication in a patient with PV and raises important questions regarding the possible contribution of IVIG therapy to intestinal ischemia.

## Case presentation

A 68-year-old man with biopsy-proven PV and a past medical history of hypertension, hyperlipidemia, type 2 diabetes mellitus, gastroesophageal reflux disease, and penetrating aortic ulcer with intramural hematoma (status post ascending aortic replacement four years prior) presented to our Veterans Hospital with two days of severe anorectal pain. He reported a five-month history of oropharyngeal ulcers and odynophagia, causing him to adopt a soft diet, followed by genital mucosal ulcerations and a cutaneous ulcer on the left arm. Tissue biopsy of these lesions indicated linear IgG deposition, diagnostic of PV [1,2].

Hospital day 1

On presentation to the emergency department of our Veterans Facility, the patient was noted to be nontoxic with a soft, nondistended, nontender abdomen but was unable to tolerate a digital rectal examination because of pain in the anorectal region, prompting consultation with General Surgery for a possible examination under anesthesia. CT of the abdomen and pelvis with IV contrast revealed rectal fecal impaction and colonic diverticulosis without diverticulitis (Figure 1). 

**Figure 1:**
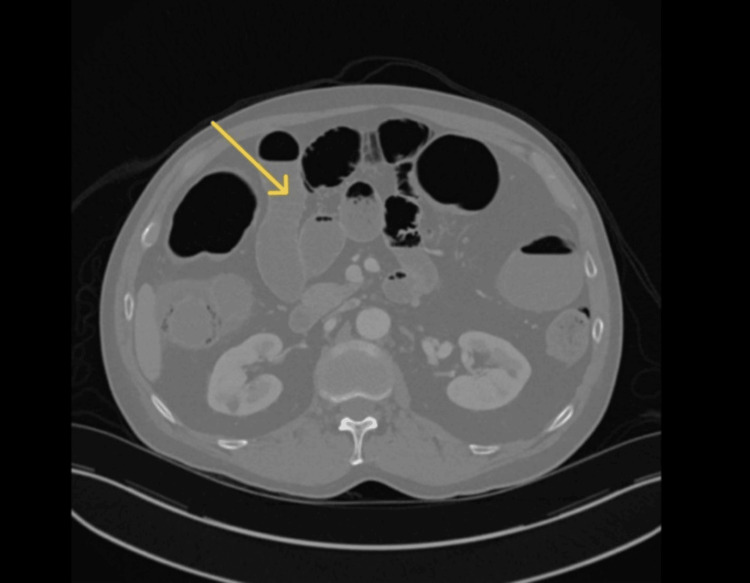
CT abdomen/pelvis with IV contrast revealed rectal fecal impaction (see arrow) and colonic diverticulosis without diverticulitis; CT on admission

He was admitted to the hospital for observation, at which point he was started on an aggressive bowel regimen to clear the fecal impaction. The next morning, he was able to tolerate a digital rectal examination, which demonstrated no significant abnormalities. Thus, continued observation was felt to be appropriate to ensure that this pain-free state continued. Accordingly, an examination under anesthesia was no longer needed.

Hospital day 2

On hospital day 2, because the rectal pain had significantly improved and a bedside rectal examination was normal, plans were made to advance his diet as tolerated and discharge him with an aggressive bowel regimen for stool burden if he tolerated such management throughout the day. At the time of admission, the patient had completed two outpatient doses of prednisone 60 mg daily, which was continued, and he began the first portion of a planned three-day split cycle of IVIG (55 g; 2 g/kg). Other outpatient medications included amlodipine, lisinopril, metoprolol, famotidine, pantoprazole, and atorvastatin.

Hospital day 3

He continued to do well during hospital day 2 and most of hospital day 3. However, on the evening of hospital day 3, he acutely deteriorated, developing severe abdominal pain, nausea, emesis, and inability to pass flatus. Laboratory evaluation revealed increasing leukocytosis (WBC 19,000/µL, increased from 16,400/µL the prior day). Abdominal radiography demonstrated dilated small-bowel loops and concern for obstruction.

Repeat CT of the abdomen and pelvis showed pneumatosis involving the cecum, ascending colon, and proximal transverse colon, with portal venous gas and cecal fat stranding (Figures 2-4) [4]. Physical examination demonstrated generalized abdominal tenderness with focal peritonitis in the right lower quadrant. This sudden deterioration was sufficiently concerning to warrant urgent exploratory laparotomy. 

**Figure 2:**
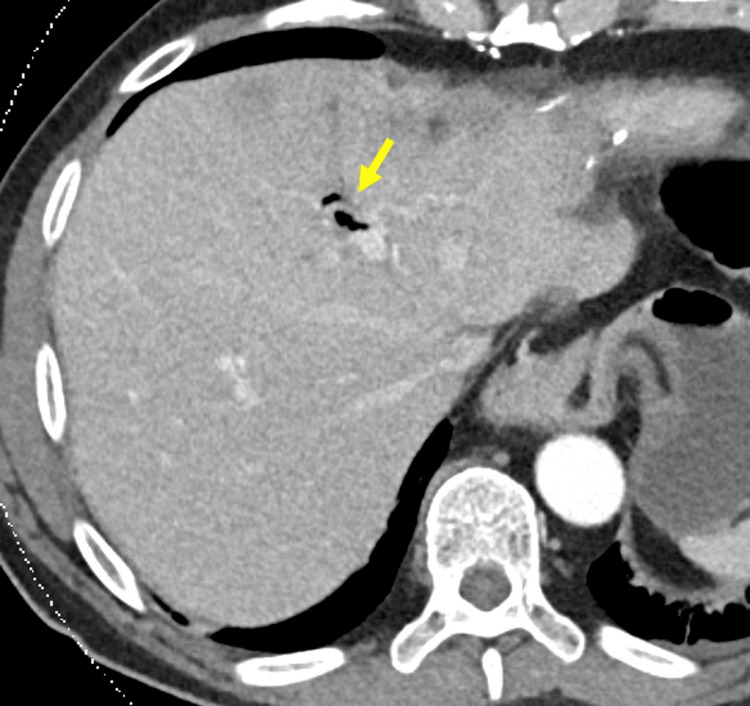
Axial CT post-contrast image demonstrates air within the portal venous system (yellow arrow)

**Figure 3:**
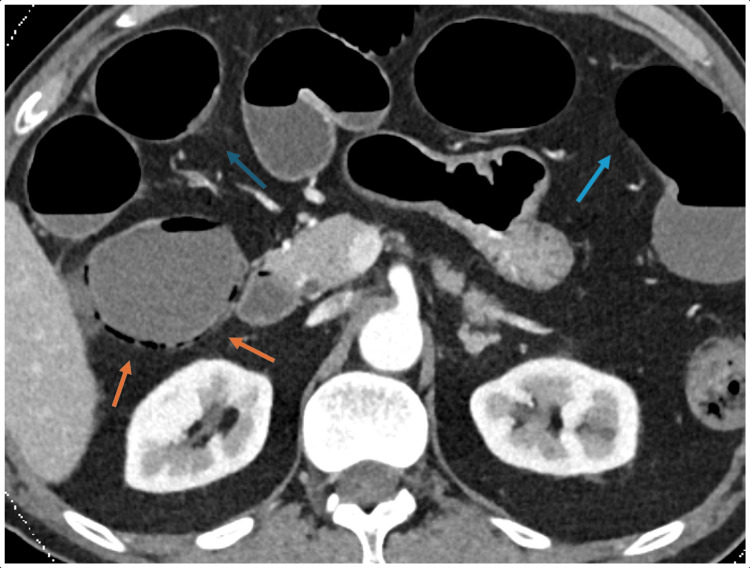
Axial CT post contrast image demonstrates pneumatosis (orange arrow), and upstream dilated small bowel loops (blue arrows)

**Figure 4:**
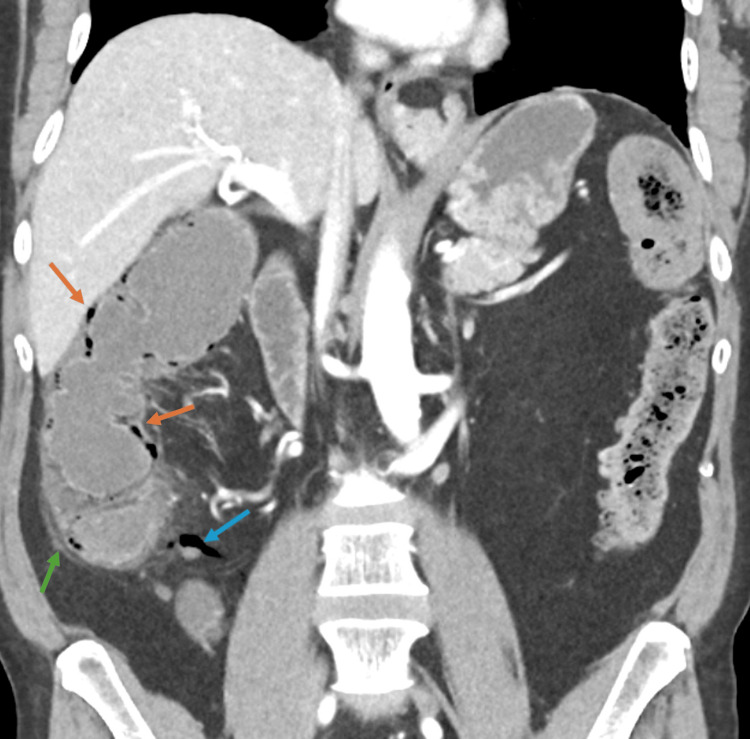
Post-contrast CT Postcontrast CT coronal image demonstrating thickening of the right-sided colon (green arrow), air within the colonic wall (pneumatosis intestinalis; orange arrows), and air within a mesenteric venous tributary (blue arrow). The findings demonstrated on this CT image are consistent with those commonly observed in acute intestinal ischemia and warrant urgent abdominal exploration.

Intraoperative findings included murky peritoneal fluid, multiple foci of colonic serosal ischemia (Figure 5) with venous thrombosis, and venous congestion of the right colon; the small bowel appeared normal.

**Figure 5:**
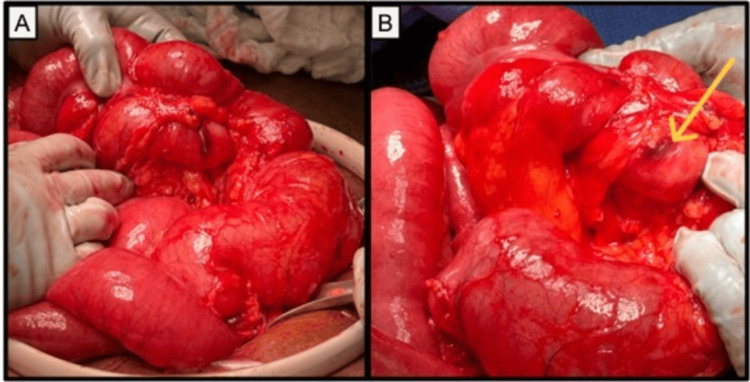
Multifocal serosal ischemia of the colon (A and B) with venous congestion (see arrow), but no evidence of perforation.

Because these abnormalities predominantly involved the cecum, an ileocecectomy with side-to-side primary anastomosis, partial omentectomy, and diverting loop ileostomy was performed. Pathologic evaluation of the resected cecum confirmed transmural ischemia without vasculitis or embolic phenomena. No hypotensive episodes were documented before this ischemic event. Its onset occurred after two doses of IVIG.

The immediate postoperative course was uneventful. By postoperative day 8, the ileostomy was consistently productive, but output sometimes exceeded 1 L daily. Loperamide was initiated to control the output, which proved successful and allowed the patient to be discharged. Although the patient had an unanticipated readmission for failure to thrive and dehydration on postoperative day 18, his fluid status and ostomy function were subsequently managed without issue, allowing discharge within 72 hours.

At his three-month postoperative clinic visit, the patient was doing well, with ostomy function enabling him to tolerate a regular diet. The ostomy was surgically closed approximately six weeks later. He has continued to do well from a surgical standpoint since that closure.

## Discussion

Involvement of the GI tract in patients with PV is distinctly uncommon. When it occurs, it is virtually always confined to the mucosa and mucous membranes. Studies have shown that the esophagus is the principal region linked with PV [5]. When this part of the GI tract is affected, it can lead to symptoms such as bleeding, emesis, swallowing difficulties, and pain. Usually, the classic blistering of the skin and oral mucous membranes, characteristic of PV, precedes esophageal involvement, but several recent reports have noted that PV may be confined to the esophagus alone [5]. Rectal involvement may also occur but is much less common than esophageal involvement. PV affecting the colon is exceedingly rare. Such involvement has generally been reported in individual case reports. In such reports, it has been difficult to determine whether there was a cause-and-effect relationship with PV or whether it was simply an incidental occurrence. Examples of colonic involvement have included unexplained hematochezia, an association with diarrhea in which usual causes were excluded, and ulcerative colitis (UC) and Crohn's disease [6]. Bullous pemphigoid has also been reported in a patient with bullous lesions of the colon [6]. There have been no reports of colonic ischemia caused by PV. Thus, our patient is the first reported case in which this association has been noted. The question naturally arises: is the association that we observed between PV and ischemic colitis simply coincidental or a true cause-and-effect relationship?

In an effort to answer this question, a number of additional research findings are relevant. In addition to isolated case reports of GI involvement in PV, a population-based study has explored a potential systemic association between pemphigus and inflammatory bowel disease [7]. In a large cross-sectional analysis of nearly 2,000 patients with pemphigus compared to matched controls, the prevalence of UC was significantly higher among those with pemphigus (0.9% vs. 0.4%, p = .004). Multivariate analysis confirmed that pemphigus was independently associated with UC (odds ratio 1.9, 95% CI 1.1-3.3), with the association being particularly strong in younger patients [7]. These findings suggest that pemphigus may predispose individuals to autoimmune inflammation of the GI tract beyond the mucocutaneous surfaces, warranting further research into shared immunologic mechanisms.

Recent evidence has also expanded our understanding of PV and related autoimmune blistering diseases through the lens of the gut-skin axis [8]. Studies suggest that alterations in the gut and skin microbiota may contribute to disease pathogenesis. In particular, dysbiosis, characterized by increased Firmicutes and depletion of beneficial probiotic species, may contribute to the pathogenesis of blistering diseases. This microbial imbalance may influence immune regulation and epithelial integrity, predisposing individuals to autoimmune activity within both cutaneous and GI tissues. Although current studies are limited, these findings highlight the microbiome as a potential modifiable risk factor and therapeutic target in PV and bullous pemphigoid [8].

Of further note, metagenomic and metabolomic analyses of stool samples have provided additional insight into the gut-skin axis in pemphigus [9]. In a comparative study of stool samples from pemphigus patients and healthy controls, investigators observed reduced gut microbial richness and diversity in pemphigus patients, with a marked decrease in Firmicutes and an increase in Proteobacteria. Beneficial butyrate-producing and anti-inflammatory bacteria were significantly depleted, and this depletion was correlated with disease activity markers (Dsg1/3 titers, PDAI scores). Metabolomic profiling revealed distinctive differences between healthy controls and PV patients, including elevated phosphatidylethanolamine (PE 18:3/14:1) and reduced levels of retinol, flavonoids, and amino acids in PV patients [9]. As described above, these findings further link microbial dysbiosis to metabolic disruption. Such findings suggest that alterations in gut microbial composition and metabolites may be connected to immune dysregulation and the pathogenesis of PV.

Despite these intriguing GI findings in PV, no direct association between PV and intestinal ischemia has been reported in the literature to date that would account for the findings observed in our patient. Thus, other factors may be involved, such as the agents used to treat PV. One such agent in our patient is IVIG. Relevant to this issue are the studies of Figueras-Aloy and colleagues [10]. These investigators conducted a 16-year retrospective review of 492 neonates with isoimmune hemolytic jaundice, 167 of whom received IVIG. They observed a markedly increased incidence of necrotizing enterocolitis (NEC) among the IVIG-treated group (6%) compared to the non-IVIG group (0.3%), identifying IVIG as an independent risk factor with an odds ratio of 31.66. The proposed mechanisms for this observation include IVIG-induced alterations in intestinal microcirculation, hyperviscosity, and immune-mediated endothelial injury, which may predispose the bowel to hypoperfusion and subsequent ischemia. In contrast, a more recent retrospective cohort study by Li and colleagues [11], which analyzed 1,259 infants with hemolytic disease of the newborn, failed to demonstrate a statistically significant relationship between IVIG exposure and NEC, thereby highlighting the conflicting nature of the available data. Of further consideration, corticosteroids, a mainstay of PV management and also used in our patient, are well recognized to impair wound healing, increase susceptibility to infection, and contribute to anastomotic breakdown in the surgical setting. However, despite the widespread use of corticosteroids in autoimmune and inflammatory diseases, there is no compelling evidence directly linking corticosteroid therapy to colonic ischemia.

So what can we conclude from these various observations? It would seem that the best conclusion that can be reached is that the ischemic insult in our patient may represent a multifactorial process in which the combined effects of underlying cardiovascular disease, which in this patient included an ulcerated aortic plaque, nutritional compromise, high-dose corticosteroids, and IVIG administration contributed to a state of mucosal vulnerability. Taken together, our case adds to the growing body of literature suggesting that IVIG may, in rare circumstances, predispose patients to intestinal ischemic complications. While causality cannot be established from a single report, this case underscores the need for vigilance in monitoring GI symptoms in patients receiving IVIG, particularly those with preexisting vascular comorbidities. Gastroenterological evaluation may be advisable in patients with PV when nutritional deficiencies are noted. Further prospective studies are needed to clarify whether IVIG independently increases the risk of intestinal ischemia, to delineate the underlying mechanisms, and to guide the risk-benefit analysis of immunosuppressive strategies in patients with PV.

## Conclusions

The case presented in this report underscores the importance of vigilance for abdominal symptoms in patients with PV receiving IVIG, particularly those with vascular comorbidities. Early recognition and prompt surgical intervention remain critical. While the temporal association between PV, IVIG, and intestinal ischemia raises suspicion for a causal relationship, current evidence remains largely circumstantial. Similar uncertainty exists in broader research on pemphigus vulgaris, where GI and microbiome abnormalities have been linked to the disease but have not been definitively proven to cause its manifestations. These findings highlight the complex interplay among immune dysfunction, treatment effects, and comorbid conditions in PV. Further studies are clearly warranted to clarify whether IVIG contributes independently to intestinal ischemia or whether this complication represents the intersection of autoimmune dysregulation, treatment effects, and vascular vulnerability in pemphigus vulgaris. On the basis of these conclusions, we believe that the learning points listed below are especially relevant.
